# FLIP_L_ is critical for aerobic glycolysis in hepatocellular carcinoma

**DOI:** 10.1186/s13046-016-0358-3

**Published:** 2016-05-13

**Authors:** Shixiong Lei, Jiandong Yang, Chong Chen, Jiachen Sun, Liu Yang, Haili Tang, Tao Yang, An Chen, Huadong Zhao, Yan Li, Xilin Du

**Affiliations:** Department of General Surgery, Tangdu Hospital, The Fourth Military Medical University, 1 Xinsi Road, Xi’an, 710032 Shaanxi China; Department of Biochemistry and Molecular Biology, The Fourth Military Medical University, 169 Changle West Road, Xi’an, Shaanxi 710032 China; Department of General Surgery, 210 Hospital of Chinese People’s Liberation Army, Dalian, 116021 China

**Keywords:** Hepatocellular carcinoma, Cellular FLICE-like inhibitory protein, Sodium-glucose cotransporter 1, Aerobic glycolysis, Glucose uptake

## Abstract

**Background:**

Tumor cells use aerobic glycolysis to rapidly generate ATP and growth substrate which expenses a large amount of glucose. However, how tumor cells take in enough glucose from the tumor microenvironment of insufficient blood supply remains poorly understood. The cellular FLICE-like inhibitory protein (FLIP), a cell apoptosis inhibiting molecule, is highly expressed in hepatocellular carcinoma (HCC) and is implicated in HCC development.

**Methods:**

The effects of FLIP_L_ (the long form of FLIP) on aerobic glycolysis and glucose uptake were assessed in HCC cells and xenograft tumors. The correlations between FLIP_L_ expression and sodium/glucose cotransporter 1 (SGLT1) expression in clinical HCC tissues were analyzed. The consequences of FLIP_L_-induced regulation of SGLT1 at the transcription and translation levels and the interaction between FLIP_L_ and SGLT1 were examined. FLIP_L_-mediated tolerance upon glucose limitation and its mechanism were detected.

**Results:**

We report a novel role for FLIP_L_ in promoting the aerobic glycolysis of HCC cells. FLIP_L_ overexpression induced a significant increase in cell aerobic glycolysis indexes including glucose uptake, glucose consumption, and lactate production. FLIP_L_ co-localized and interacted with SGLT1, a major active glucose transporter in HCC cells. FLIP_L_ increased the stability of SGLT1 protein by inhibiting its ubiquitination and degradation. The expression level of FLIP_L_ was positively correlated with the expression level of SGLT1 in 79 HCC tissues from surgical operation. Furthermore, FLIP_L_ increased cell tolerance ability and decreased cell apoptosis to low glucose by regulating SGLT1.

**Conclusions:**

Our results indicate that FLIP_L_ plays an essential role in HCC aerobic glycolysis and that SGLT1 is required for FLIP_L_-modulated tumor proliferation under low glucose conditions. Targeting the actions of FLIP_L_ in cell glucose-dependent aerobic glycolysis may provide an attractive strategy for therapeutic intervention in HCC.

**Electronic supplementary material:**

The online version of this article (doi:10.1186/s13046-016-0358-3) contains supplementary material, which is available to authorized users.

## Background

Hepatocellular carcinoma (HCC) is a highly aggressive malignant tumor worldwide. Due to the resistance of HCC to conventional therapies, such as radiotherapy and chemotherapy, hepatic resection remains the most effective method for treating HCC [1, 2]. However, many patients have already lost the opportunity for hepatic resection by the time that HCC is diagnosed. Therefore, continued efforts are needed to find new targets for HCC treatment.

Tumor cells convert most glucose into lactate regardless of whether oxygen supply is sufficient. This aerobic glycolytic metabolism is known as the Warburg effect [3]. Tumor cells utilize aerobic glycolysis to rapidly generate ATP and other substrates to meet the need of tumor growth [4]. In view of the fact that aerobic glycolysis is much less efficient than oxidative phosphorylation for generating ATP [5], tumor cells need to increase glucose absorption to meet the high requirement for glucose metabolism [6]. Multiple oncogenes and tumor suppressor genes participate in the glucose uptake and metabolism processes, including PI3K, AKT, AMPK, p53, c-Myc, and HIF-1α [7, 8]. These regulators increase glucose uptake by enhancing the transcription and membrane translocation of glucose transporters in tumor cells. There are two classes of glucose transporters in humans, the facilitated transporters (GLUTs) and the active transporters (SGLTs). Previous studies have revealed that ectopic expression of glucose transporters in many tumors is beneficial to cell proliferation and survival under low glucose condition [9]. It has been reported that there is abnormal GLUT1 expression in HCC [10]. Despite glucose transporters is significant in tumor aerobic glycolysis, the key molecules and precise mechanisms controlling the glucose transporters are still largely unknown.

Cellular FLICE-like inhibitory protein (FLIP) is a potential oncogene in HCC, which highly homologous to caspase-8 containing two death effector domains [11]. The human *FLIP* gene is located on chromosome 2q33-q34. Three splice variants of the FLIP protein have been identified, a 55 kDa long form FLIP (FLIP_L_), a 27 kDa short form FLIP (FLIP_S_) and a 25 kDa FLIP Raji (FLIP_R_) [12]. These isoforms perform different cellular functions [12], with FLIP_L_ being the most well-studied isoform. Elevated FLIP_L_ is observed in many cancers, including HCC, malignant melanoma, breast cancer, prostate cancer, bladder cancer, and non-Hodgkin lymphoma [13]. FLIP_L_ has been shown to be a multifunctional protein involved in death receptor-mediated apoptosis, the NF-κB pathway [14, 15], necrosis [16], autophagy [17], inflammation [18], the ubiquitin-proteasome system [19], and endoplasmic reticulum morphology [20]. Recently, FLIP_L_ was shown to be up-regulated following the disruption of glycolysis with 2-deoxy-D-glucose (2-DG) [21]. However, whether and how FLIP_L_ participates in glucose metabolism in cancer cells is unclear.

In the current study, we first demonstrated the function of FLIP_L_ in facilitating HCC cells’ aerobic glycolysis by modulating SGLT1-mediated glucose uptake. We found SGLT1 is required for FLIP_L_ induced cell aerobic glycolysis and survival to low glucose conditions. We also extended our findings to clinical HCC patients. In 79 HCC cases, FLIP_L_ expression level was positively correlated with SGLT1 expression level. Therefore, our results suggest that FLIP_L_ is a potential therapeutic target for HCC [22].

## Methods

### Cells and reagents

HepG2, MHCC97-H, Huh-7, SMMC-7721, BEL-7704 HCC, and HEK293 cell lines were obtained from the Department of Biochemistry and Molecular Biology (The Fourth Military Medical University) and cultured in a humidified incubator under 5 % CO_2_ in Dulbecco’s modified Eagle’s medium (DMEM) (Life Technologies, Carlsbad, CA, USA) supplemented with 10 % fetal bovine serum (FBS), 2 mM L-glutamine, 100 U/ml penicillin, and 100 mg/ml streptomycin at 37 °C. The proteasome inhibitor, MG132, was from Sigma-Aldrich (Shanghai, China). Mouse anti-GLUT1, rabbit anti-SGLT1 and mouse anti-FLIP antibodies were obtained from Abcam (New Territories, HK). Rabbit anti-FLIP and mouse anti-HA-Tag antibodies were from Cell Signaling Technology (Danvers, MA). 2-NBDG (2-[N-(7-Nitrobenz-2-oxa-1,3-diazol-4-yl)amino]-2-deoxy -D-glucose) was purchased from Cayman Chemical Company (Michigan, US).

### Cell transfections

FLIP_L_ expression plasmid (Flag-FLIP_L_), FLIP_S_ expression plasmid (Flag-FLIP_S_), SGLT1 expression plasmid (Myc-SGLT1), HA-ubiquitin expression plasmid, shRNA targeting FLIP_L_, and shRNA targeting SGLT1 were constructed by Jikai biotechnology (Shanghai, China). Cells (1 × 10^6^) were seeded into six-well plates and transfected using Lipofectamine 2000 (Invitrogen Life Technologies, Carlsbad, USA) according to the manufacturer’s instructions. The target sequences of shRNA of FLIP_L_ and SGLT1 are 5’ CAGAATAGACCTGAAGACA3’ and 5’ CTTCCGCATCCAGGT CAAT3’, respectively.

### Real-time PCR

RNA was reverse transcribed with the PrimeScript™ 1st Strand cDNA Synthesis Kit (Takara, Dalian, China) according to the manufacturer's instructions. Real-time PCR was performed using SYBR Premix Ex Taq™ II (Takara, Dalian, China). The relative gene expression was calculated using the 2^−ΔΔCt^ method, in which Ct represented the threshold cycle. β-actin RNA was used as the reference. The primers were used as following. FLIP_L_: Forward 5’GGCTCCCCCTGCATCAC3’, Reverse 5’ TTTGGCTT CCTGCTAGATAAGG3’; SGLT1: Forward 5’ATGGACAGTAGCACCTTGAGCC3’; Reverse 5’TAGCCCCAGAGAAGATGTCTGC3’; GLUT1: Forward 5’ AAGTCTCC TTTACCCACATCC 3’; Reverse 5’ GAGTGTCCGTGTCTTCTTGAGT 3’.

### Immunoblot analysis

Cells were harvested and lysed with RIPA buffer (0.15 M NaCl, 1 % NP40, 0.01 M deoxycholate, 0.1 % SDS, 0.05 M Tris-HCl pH 8.0, 1 mM sodium orthovanadate, 1 mM phenylmethylsulfonyl fluoride, 10 mg/ml aprotinin, 10 mg/ml pepstatin and 10 mg/ml leupeptin). Protein concentrations were determined using the BCA Protein Assay kit (Beyotime, China). The total protein was electrophoresed on 8 or 12 % SDS-PAGE gels and transferred to polyvinylidene fluoride membranes. Membranes were blocked with 5 % nonfat dry milk or BSA in TBST buffer (20 mM Tris-HCl pH 7.4, 8 g/L NaCl and 0.1 % Tween 20) for 1 h at room temperature, and then incubated with the indicated primary antibody in TBST buffer containing 2 % BSA at 4 °C overnight. The membrane was then probed with the appropriate horseradish peroxidase-conjugated secondary antibody at room temperature for 1 h, and the proteins were detected with a chemiluminescence imaging analysis system.

### Glucose uptake assay in vitro

Cells were plated at 1×10^6^/well in 6-well plates and cultured for 24 h. After the specified treatments, cells were refreshed with serum-starved (0.1 % FBS) and glucose-free DMEM. 16 h later, cells were grown in the presence of 50 mM 2-NBDG for 30 or 60 min, respectively, and glucose uptake was quantified using FACS analysis.

### Tumorigenesis and glucose uptake assay in vivo

5×10^6^ of HepG2 cells were injected into the hind legs of athymic male nude mice (8 weeks, seven mice per group). The tumor size was measured weekly with a caliper, and the tumor volume was determined with the standard formula: 0.5×length×width^2^. The tumor growth curve was then derived from these data. ^18^F-FDG was injected into the mice via a tail vein and PET/CT images were collected on a nanoScan PET/CT system (Mediso, Budapest, Hungary).

### Lactate production assay

1×10^5^ cells were plated in 6-well plates and cultured overnight. Culture medium was removed from cells and the lactate concentration was determined using the Lactate Kit (Nangjing Jiancheng Bioengineering, Nanjing, China) according to manufacturer’s protocol. Cells were then harvested, stained with trypan blue, and the number of viable cells was counted using a hemocytometer. The rate of lactate production was determined (lactate production rate=lactate concentration/cells/time) and normalized to the rate detected in the control group.

### Glycolysis assay

Extracellular acidification rate (ECAR) was conducted using a Seahorse XF24 Analyzer. 20,000 cells were seeded into Seahorse 24-well microplates and cultured at 37 °C with 5 % CO_2_ for 24 h. Culture medium was replaced with 700 μl of assay medium, composed of DMEM without FBS and sodium bicarbonate, and cells were then incubated at 37 °C without CO_2_ for 1 h. To measure the glycolytic capacity of the cells, additions were injected to the wells at final concentrations of 11 mM glucose, 1 μM oligomycin, and 20 mM 2-DG.

### Immunofluorescence assay

Cells were fixed in a freshly prepared 4 % paraformaldehyde, rinsed with 0.1 % PBS and permeabilized with Triton X-100. Cells were then incubated with horse serum in PBS to block nonspecific binding. After being washed with PBS, cells were incubated overnight at 4 °C with mouse anti-FLIP antibody (1:200), rabbit anti-SGLT1 antibody (1:200). Subsequently, fluorescein isothiocyanate-conjugated anti-mouse antibody (diluted 1:400; Sigma-Aldrich) or cyanine 3-conjugated anti-rabbit antibody (diluted 1:400; Sigma-Aldrich) were incubated with the cells at room temperature for 2 h. The isotype mouse and rabbit IgG were used as negative controls. Fluorescence detection was performed using a laser confocal microscope (Nikon, US) after cell nucleus counterstaining with 4’, 6-diamidino-2-phenylindole.

### Immunoprecipitation

Cells were incubated with 1 ml of lysis buffer containing (50 mM HEPES, pH 7.4; 150 mM NaCl; 0.1 % Triton X-100; 1.5 mM MgCl_2_; 1 mM EDTA; and 1 mM phenylmethylsulfonyl fluoride), as well as protease inhibitors for 30 min at 4 °C. The insoluble fraction was eliminated through centrifugation at 12,000 rpm for 30 min at 4 °C. The lysates were incubated with the antibody after centrifugation and protein A/G-conjugated sepharose (Santa Cruz Bio Technology, USA) at 4 °C overnight. 20 μl of protein was saved to quantify the total amount of protein loaded. Beads were washed four times with lysis buffer. Proteins were eluted in SDS-PAGE sample buffer and separated by SDS-PAGE for immunoblot analysis.

### Cell proliferation assay

Cells were seeded into 96-well cell culture plates. Each well contained 1×10^3^ cells in 100 μl culture media. After the specified treatments, the medium in each well was replaced with 100 μl fresh medium with 10 % CCK8 (Dojindo, Japan), and the cells incubated at 37 °C for additional 2 h. The absorbance was measured at 450 nm wavelength.

### Cell apoptosis assay

Cells were cultured in MEM containing low glucose (0.75 mM) or high glucose (25 mM) for 48 h after cell transfection. For Annexin staining, cells were trypsinized and resuspended in 1 ml Annexin-binding buffer (BD Biosciences, US) to which was added 5 μl Annexin V-FITC (BD Biosciences, US). After incubation in the dark at room temperature for 15 min, 50 μl PI (Sigma, US) was added to discriminate dead cells and the samples were analyzed on a FACS Caliber flow cytometer.

### Tissue samples and study cohort and immunohistochemistry (IHC)

All patients from whom we obtained the 79 pairs of HCC and adjacent normal liver tissue specimens provided their full consent to participate in the study at the Xijing Hospital of the Fourth Military Medical University (Xi’an, China). IHC was performed using the avidin-biotin-peroxidase complex method. All sections were deparaffinized in xylenes and dehydrated through a gradient concentration of alcohol before endogenous peroxidase activity was blocked using 0.5 % H_2_O_2_ in methanol for 10 min. After nonspecific binding was blocked, the slides were incubated with FLIP_L_ antibody (1:200) or SGLT1 antibody (1:200) in phosphate-buffered saline (PBS) at 4 °C overnight in a humidified container. Biotinylated goat anti-rabbit immunoglobulin G (IgG) (1:400; Sigma-Aldrich) was incubated with the sections for 1 h at room temperature and detected using a streptavidin-peroxidase complex. The brown color indicative of peroxidase activity was developed by incubation with 0.1 % 3, 3-diaminobenzidine in PBS with 0.05 % H_2_O_2_ for 5 min at room temperature. An immunoreactivity score system was provided on the basis of the proportion and intensity of positively stained cancer cells. The extensional standard used was as follows: (1) the number of cells with positive staining (0–5 %: 0; 6–25 %: 1; 26–50 %: 2; 51–75 %: 3; and>75 %: 4) and (2) the staining intensity (colorless: 0; pallide-flavens: 1; yellow: 2; brown: 3). We multiplied the numbers scored (1) and (2) from the extensional standard, and the staining grade was stratified as absent (0 score), weak (1–4 score), moderate (5–8 score) or strong (9–12 score). Two pathologists blinded to the clinicopathologic information and outcome of the patients scored the multiple tissue arrays. Tumors with moderate or strong immunostaining intensity were classified as staining positive (+), whereas tumors with absent were classified as staining negative (−).

### Statistical analysis

Data are reported as the mean ± S.D. or S.E.M. analyses were performed using GraphPad Prism software. The significance of differences was determined by one-way analysis of variance followed by Scheffe’s post hoc test, independent samples t-test or Student’s t-test. Associations between protein expression and categorical variables were analyzed using the χ^2^ test or Fisher’s exact test as appropriate. *P* < 0.05 was considered statistically significant.

## Results

### FLIP_L_ facilitates aerobic glycolysis by promoting glucose uptake

**Fig. 1:**
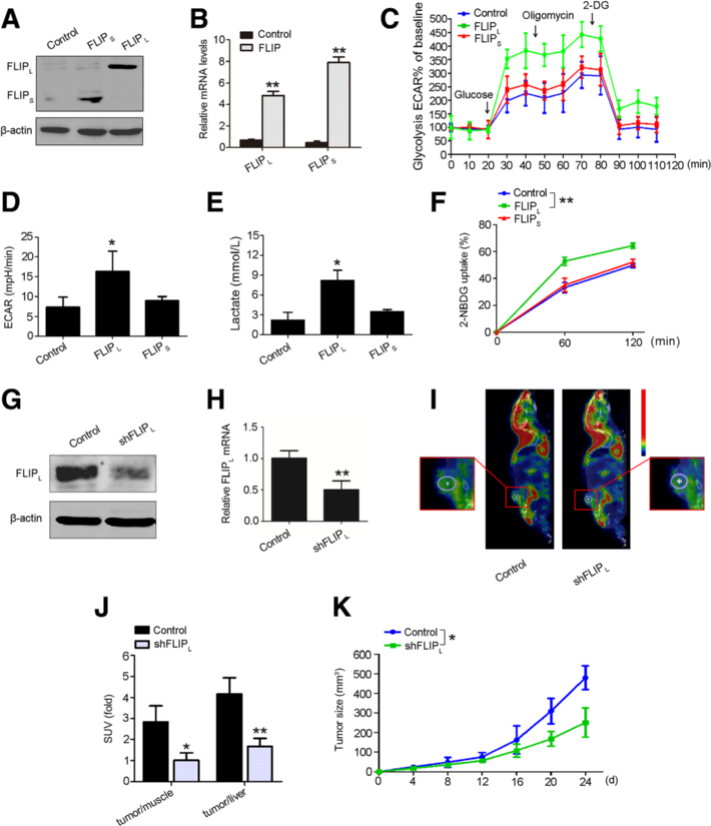
FLIP_L_ promotes aerobic glycolysis by up-regulating glucose uptake. **a** and **b** HepG2 cells were transfected with FLIP_L_ or FLIPs overexpression vectors. Expression was detected 48 h after transfection in protein and mRNA levels. Data are the mean ± S.E.M. from three independent experiments. **p < 0.01 versus the control group. Cell ECAR in **c** and **d** was measured by the glycolysis stress test in HepG2 cells with FLIP_L_ or FLIP_S_ overexpression. Data represent the mean ± S.E.M. from three independent experiments, each performed in quadruplicate. *p < 0.05 versus the control group. **e** Lactate production was measured in HepG2 cells with FLIP_L_ or FLIP_S_ overexpression. Data represent the mean ± S.E.M. from three independent experiments, each performed in quadruplicate. *p < 0.05 versus the control group. **f** Glucose uptake was measured in HepG2 cells with FLIP_L_ or FLIP_S_ overexpression. Cells were grown in the presence of the fluorescent analog 2-NBDG for various time points, and glucose uptake was quantified using FACS analysis. Data represent the mean ± S.E.M. Similar results were obtained from three independent experiments. **p < 0.01 versus the control group. **g** and **h** HepG2 cells were transfected with shFLIP_L_ vector. Expression was detected 48 h after transfection in protein and mRNA levels. Data are the mean ± S.E.M. from three independent experiments. **p < 0.01 versus the control group. **i** Representative PET/CT images in nude mice bearing liver tumors with control or shFLIP_L_ plasmid treatment. Yellow dotted lines indicate area of the liver tumors. **j** Glucose uptake in HCC was normalized with muscle or liver tissues in each mouse (*n*=8). Data are presented as the mean ± S.D. *p < 0.05, **p < 0.01 versus control. **k** Tumor development in nude mice bearing with FLIP_L_-silent HepG2 cells (*n*=8). Data are presented as the mean ± S.D. *p < 0.05 versus control

To investigate whether FLIP could affect aerobic glycolysis in HCC, FLIP_L_ and FLIP_S_, the two main isoforms of FLIP, were overexpressed in HepG2 cells (Fig. 1a, b). Overexpressed FLIP_L_ significantly increased aerobic glycolytic capacity in response to glucose stimulation compared with the control group, but overexpressed FLIP_S_ had no effect on glycolysis (Fig. 1c, d). We also found that FLIP_L_, but not FLIP_S_, induced lactate production (Fig. 1e), which was consistent with the ECAR results. These findings suggest that FLIP_L_ is involved in aerobic glycolysis in HCC.

Glucose uptake is a key step in aerobic glycolysis, which is critical for cancer cell survival. Most cancer cells generate high levels of ATP through increasing the glucose uptake [23]. Therefore, we examined the effect of FLIP on glucose uptake. Ectopic expression of FLIP_L_ significantly induced glucose uptake in HepG2 cells, but glucose uptake was not altered in cells overexpressing FLIP_S_ (Fig. 1f). Moreover, to evaluate the influence of FLIP_L_ on glucose uptake and HCC progression in vivo, nude mice bearing FLIP_L_ down-regulated HepG2 cells (Fig. 1g, h) were imaged by ^18^F-FDG PET/CT scanning. The scan images demonstrated that ^18^F-FDG accumulation was notably decreased with the deletion of FLIP_L_ (Fig. 1i, j). We also analyzed the effect of FLIP_L_ on HCC development by continuously measuring the size of tumors in the xenograft mouse model. As shown in Fig. 1k, the growth of transplanted tumors with FLIP_L_ silenced was significantly slower, compared with the control group. These results indicated that FLIP_L_ promotes tumor cell survival and aerobic glycolysis by increasing glucose uptake both in vivo and in vitro.

### FLIP_L_ expression positively correlated with SGLT1 expression in hepatic carcinoma

**Fig. 2:**
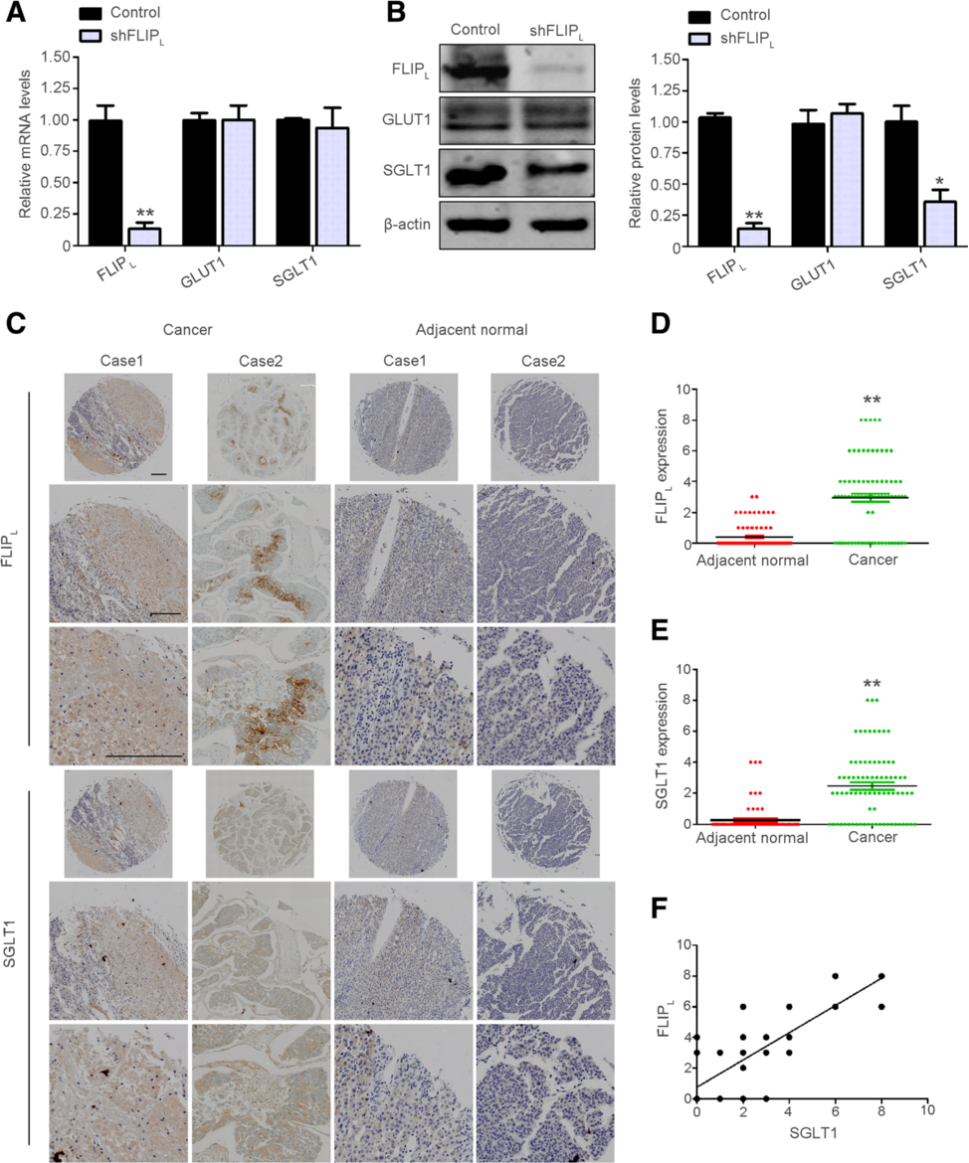
FLIP_L_ expression positively correlated with SGLT1 expression in HCC. GLUT1 and SGLT1 expression were determined by real-time PCR (**a**) and immunoblot analysis (**b**) in HepG2 cells with FLIP_L_ knockdown treatment. Left panel: Immunoblotting. Right panel: Densitometric quantification of the data in **b**, normalized to β-tubulin. Data are the mean ± S.E.M. from three independent experiments. *p < 0.05, **p < 0.01 versus the control group. **c** IHC staining of FLIP_L_ and SGLT1 in adjacent normal and liver cancer samples from patients at different stages. Case 1 is from a stage II patient, and Case 2 is from a stage I patient. Bar = 200 μm. (**d**) and (**e**) Quantification of the expression levels of FLIP_L_ and SGLT1 in human HCC samples. **p < 0.01 versus adjacent normal tissues. **f** Correlation between FLIP_L_ and SGLT1 expression with linear regression and Pearson’s correlation significance (*p* < 0.01)

Glucose is transported into cells by two families of transporters, the facilitative-type glucose transporters and the active-type glucose transporters [24]. GLUT1 and SGLT1, two well-studied glucose transporters in the respective families, are highly expressed in several cancers and participate in cellular glucose uptake. To determinate the mechanisms by which FLIP_L_ promotes the aerobic glycolysis and survival of HCC cells, we measured the GLUT1 and SGLT1 mRNA and protein levels after shRNA-mediated depletion of FLIP_L_ expression in HepG2 cells. Although the mRNA level of SGLT1 did not change, its protein expression was notably decreased following FLIP_L_ loss. However, neither mRNA nor protein levels of GLUT1 altered with the down-regulation of FLIP_L_ (Fig. 2a, b). These findings suggested that SGLT1, but not GLUT1, is involved in FLIP_L_-mediated HCC glucose uptake.

Dysregulated expression of SGLT1 has been identified in various human cancers. However, the expression status of SGLT1 in HCC has not been investigated. To assess SGLT1 protein expression levels and its potential association with FLIP_L_ in HCC, we compared 79 pairs of HCC and adjacent normal tissue samples using immunohistochemistry (IHC). The results showed that SGLT1 and FLIP_L_ were highly expressed in liver cancer tissue compared with adjacent normal liver tissue (Fig. 2c). In normal versus liver cancer tissue, FLIP_L_ levels were 0.41 versus 2.97 (Fig. 2d), and SGLT1 were 0.28 versus 2.47 (Fig. 2e). Moreover, in these 79 pairs of HCC and adjacent normal tissue samples, SGLT1 and FLIP_L_ expression were correlated with tumor stage (Tables 1 and 2) in liver cancer tissues, but the correlation between the expression and sex or age was not statistically significant in both liver cancer tissues and their adjacent normal specimens (Tables 1 and 2, Additional file 1: Table S1 and Table S2).

**Table 1:** Statistical results of FLIP_L_ expression in 79 liver cancer specimens

	N	-	+to+++	*P*-value
Total	79	24	55	
Sex				0.605*
Male	66	20	46	
Female	13	4	9	
Age at diagnosis, years				0.437**
<60	61	18	35	
≥60	18	6	20	
TNM stage				0.015**
I	22	12	10	
II	40	11	29	
III	17	1	16	

*Abbreviation*: *TNM* tumor node metastasis. *P-value when expression levels were compared using Fisher’ exact test; **P-value when expression levels were compared using the Kruskal-Wallis test

**Table 2:** Statistical results of SGLT1 expression 79 liver cancer specimens

	N	-	+to+++	*P*-value
Total	79	23	56	
Sex				0.490*
Male	66	21	45	
Female	13	2	9	
Age at diagnosis, years				0.769*
<60	61	17	44	
≥60	18	6	12	
TNM stage				0.046**
I	22	9	13	
II	40	13	27	
III	17	1	16	

*Abbreviation*: *TNM* tumor node metastasis. *P-value when expression levels were compared using Fisher’ exact test; **P-value when expression levels were compared using the Kruskal-Wallis test

Because knocking down FLIP_L_ expression led to the suppression of SGLT1 protein levels, we speculated whether there is a correlation between the protein levels of FLIP_L_ and SGLT1. We analyzed the correlation between FLIP_L_ and SGLT1 expression using spearman analysis. The expression of FLIP_L_ was demonstrated to positively correlate with SGLT1 expression (Fig. 2f), suggesting that FLIP_L_ is involved in the post-transcriptional regulation of SGLT1.

### FLIP_L_ interacts with SGLT1

**Fig. 3:**
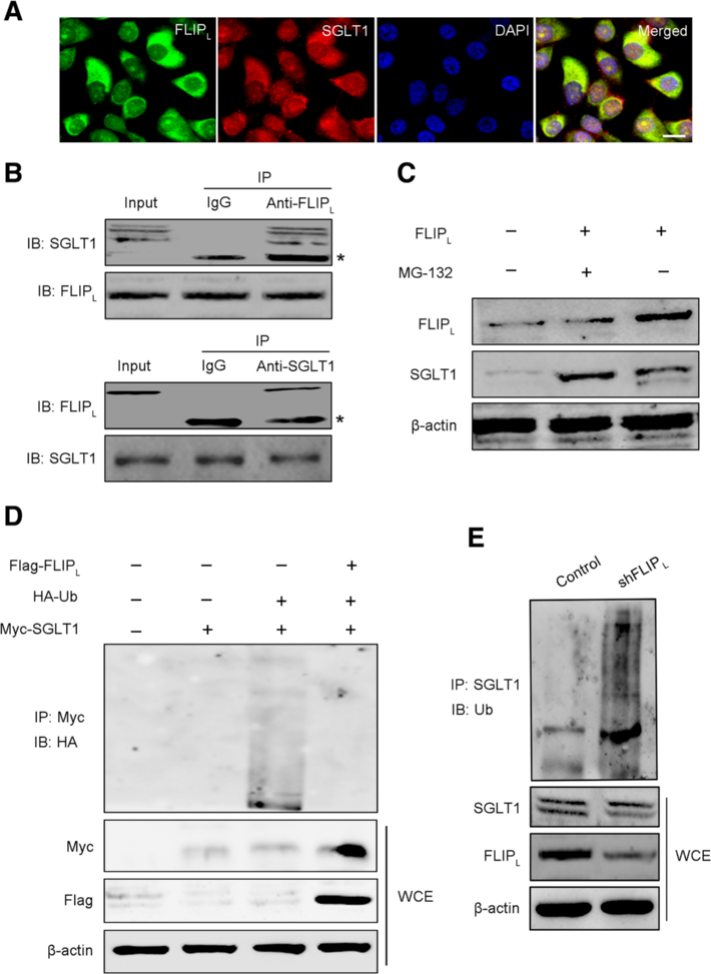
FLIP_L_ inhibits the ubiquitination and degradation of SGLT1 by interacting with SGLT1. **a** The localization of FLIP_L_ and SGLT1 were determined in HepG2 cells by laser confocal scanning microscopy. Green fluorescence indicated FLIP_L_ expression, red fluorescence indicated SGLT1 expression, and blue fluorescence indicated nuclear staining with DAPI. Bar = 20 μm. **b** HepG2 cells were harvested for co-immunoprecipitation assays followed by immunoblot (IB) analysis. Immunoprecipitation (IP) assays were performed with whole-cell lysates (WCE) pretreated with A/G–conjugated Sepharose beads. WCE were probed for input. The antibodies used for IP and IB are as indicated. **c** SGLT1 expression was determined in HepG2 cells with FLIP_L_ overexpression for 48 h and/or 5 μM MG132 for another 4 h. **d** HEK293 cells were transfected with hemagglutinin (HA)-ubiquitin plasmid, Flag-FLIP_L_ plasmid, and Myc-SGLT1 plasmid for 48 h. Subsequently, the cell lysates were collected and analyzed by IP and IB. **e** HepG2 cells were transfected with HA-ubiquitin and shFLIP_L_ plasmids for 48 h, and cell lysates were collected and analyzed by IP and IB

Given that FLIP_L_ could regulate the protein level of SGLT1, but not the SGLT1 transcript, we investigated whether the FLIP_L_ protein interacts with the SGLT1 protein. To address this question, we examined the subcellular distribution of FLIP_L_ and SGLT1 in HepG2 cells using immunofluorescence. Both FLIP_L_ and SGLT1 were predominantly localized in the cytoplasm and there was a strong co-localization of the two proteins (Fig. 3a). The co-localization of FLIP_L_ and SGLT1 prompted us to further analyze whether FLIP_L_ could bind with SGLT1. Co-immunoprecipitation was performed using lysates prepared from HepG2 cells, and showed that endogenous FLIP_L_ interacted with endogenous SGLT1, and vice versa (Fig. 3b).

### FLIP_L_ inhibits the ubiquitination and degradation of SGLT1

FLIP_L_ has been shown to up-regulate the levels of some proteins by inhibiting the ubiquitin-proteasome degradation pathway [19]. We hypothesized that FLIP_L_ stabilizes SGLT1 protein by regulating its degradation. First we determined the protein degradation pathway of SGLT1. As shown in Fig. 3c, FLIP_L_ increased the SGLT1 protein level and MG132, a proteasome inhibitor, induced further accumulation of SGLT1 protein, indicating that the degradation of SGLT1 protein occurs via a proteasome associated pathway. In addition, we examined the effect of FLIP_L_ on SGLT1 ubiquitination in HEK293 cells and HepG2 cells. In HEK293 cells, exogenous FLIP_L_ inhibited the ubiquitination of exogenous SGLT1 and increased the total levels of SGLT1 protein (Fig. 3d). Endogenous SGLT1 ubiquitination was also examined in HepG2 cells and, consistent with the results with the exogenous proteins in HEK293 cells, we found that down-regulation of the expression of FLIP_L_ led to increased SGLT1 ubiquitination and accelerated SGLT1 degradation (Fig. 3e). Collectively, these results suggested that FLIP_L_ interacts with SGLT1 in the cytoplasm and increases the stability of SGLT1 protein by inhibiting its ubiquitination and proteasome-mediated degradation. This promotes the membrane translocation and function of SGLT1.

### FLIP_L_ promotes the tolerance of HCC cells to low glucose

**Fig. 4:**
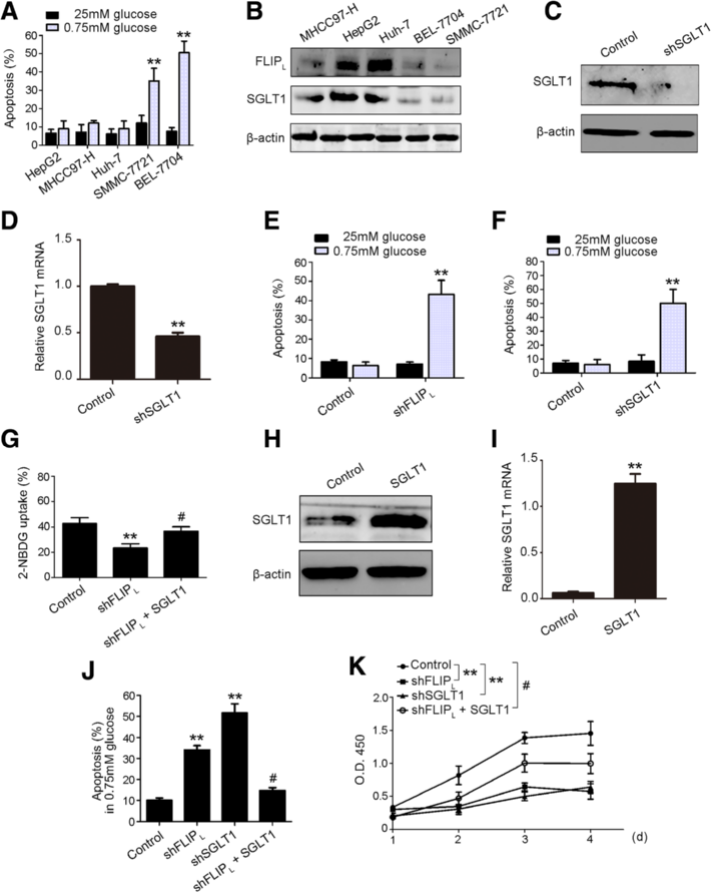
FLIP_L_ promotes low glucose tolerance in HCC cells. **a** FACS analysis was performed to determine the apoptosis of HepG2, MHCC97-H, Huh-7, SMMC-7721 and BEL-7704 cells under low glucose (0.75 mM) or normal glucose (25 mM) treatment. **b** Immunoblot was used to detect the FLIP_L_ and SGLT1 expression in MHCC97-H, HepG2, Huh-7, BEL-7704, and SMMC-7721 cell lines. **c**, **d** HepG2 cells were transfected with shSGLT1 vector. Expression was detected 48 h after transfection at protein and mRNA levels. **p < 0.01 versus control. **e** The apoptosis was measured in HepG2 cell with FLIP_L_ knockdown treatment. Cells were cultured in low glucose (0.75 mM) or normal glucose (25 mM) and the levels of apoptosis were determined by FACS analysis. **f** Apoptosis was measured in HepG2 cell with SGLT1 knockdown. Cells were cultured in low glucose (0.75 mM) or normal glucose (25 mM), apoptosis was determined by FACS analysis. **g** Glucose uptake was measured in HepG2 cell with FLIP_L_ knockdown or FLIP_L_ knockdown plus SGLT1 overexpression treatment. **h**, **i** FLIP_L_-deficient HepG2 cells were transfected with the SGLT1 overexpression vector. Expression was detected 48 h after transfection at protein and mRNA levels. **p < 0.01 versus control. **j** Apoptosis was measured in HepG2 cell with FLIP_L_ knockdown, SGLT1 knockdown, and FLIPL knockdown plus SGLT1 overexpression treatment. **k** Cell proliferation was measured by CCK8 assay in HepG2 cells with FLIP_L_ knockdown, SGLT1 knockdown, and FLIP_L_ knockdown plus SGLT1 overexpression treatment. (**a**, **e**, and **f**) Data represent the mean ± S.E.M. from three independent experiments, each performed in quadruplicate. **p < 0.01 versus 25 mM glucose group. (**g**, **i**, **j**, and **k**) Data represent the mean ± S.E.M. from three independent experiments, each performed in quadruplicate. *p < 0.05, **p < 0.01 versus control group; ^#^ *p* < 0.05 versus shFLIP_L_ group

High level of glycolysis could not only rapidly provide ATP for cancer cell proliferation but also allow cancer cells to adapt low glucose condition [9]. We investigated whether and how FLIP_L_ regulates the tolerance for low glucose of HCC cells. We first assessed the ability of several HCC cell lines to tolerate low glucose. The apoptosis levels of HepG2, MHCC97-H, Huh-7, SMMC-7721 and BEL-7704 were detected under high glucose condition (25 mM) and low glucose condition (0.75 mM). Of these cell lines, HepG2, MHCC97-H, and Huh-7 cells exhibited higher tolerance of the low glucose condition (Fig. 4a). We next examined the FLIP_L_ and SGLT1 expressions in these cell lines. FLIP_L_ and SGLT1 protein levels were higher in two of the cell lines tolerant to low glucose, HepG2 and Huh-7 cells (Fig. 4b), so we used the HepG2 cells to perform the following experiments.

To determine whether FLIP_L_ and SGLT1 affect the susceptibility of HCC cells to low glucose conditions, FLIP_L_ and SGLT1 were separately down-regulated in HepG2 cells (Figs. 1g, h, and 4c, d). Examination of cell apoptosis, as shown in Fig. 4e, f, showed that apoptosis rates were significantly increased in FLIP_L_-silenced or SGLT1-silenced HepG2 cells. In addition, we found that the loss of FLIP_L_ inhibited glucose uptake in HepG2 cells (Fig. 4g), which is consistent with the results of FLIP_L_ overexpression in Fig. 1f. We next rescued the SGLT1 expression in FLIP_L_-deficient HepG2 cells (Fig. 4h, i), and the reduced glucose uptake was substantially recovered (Fig. 4g). The increased apoptosis rate was also attenuated with the restoration of SGLT1 expression in FLIP_L_-deficient HepG2 cells (Fig. 4j). In addition, we examined the survival of HepG2 cells in a low glucose environment. FLIP_L_-deficient HepG2 cells exhibited a decreased cell proliferation rate, and rescued SGLT1 expression significantly recovered cell survival under low glucose treatment (Fig. 4k). These results demonstrated that FLIP_L_ promotes glucose uptake and the survival of HCC cells under low glucose conditions through regulation of SGLT1.

## Discussion

In the present study, we determined a previous unknown role for FLIP_L_ in HCC cells aerobic glycolysis. We have made three novel observations here. First, we demonstrated that FLIP_L_ interacted with SGLT1 to inhibit the ubiquitination of SGLT1, thereby promoting glucose uptake and glycolysis in HCC cells. Second, we found that FLIP_L_ was highly expressed in HCC tissue compared with adjacent normal tissue and that FLIP_L_ expression positively correlated with SGLT1 expression. Third, FLIP_L_ increased tumor cell tolerance to glucose limitation through the regulation of SGLT1 protein stability.

Glucose, as the main energy source of cells, is vital to tumor cell proliferation. Aerobic glycolysis is a hallmark in various tumors [25, 26]. Glucose uptake is the first step in glycolysis. HCC enhanced glucose uptake to meet the metabolic requirement of cells [27]. A previous study has shown that a glucose transporter can be a novel and attractive therapeutic target for HCC [28]. In the current study, we found that SGLT1 was highly expressed in HCC. Our findings support the notion that SGLT1 can be a promising intervention target for HCC.

Multiple molecules are involved in the regulation of tumor glucose metabolism. Investigating the factors that control cancer cell aerobic glycolysis could not only advance the knowledge of the regulation of glucose metabolism in cancer cells but also provide new approaches for cancer therapy. Many factors have been shown to regulate or affect aerobic glycolysis, oxidative phosphorylation, pentose phosphate pathway, and glutamine metabolism. The well-studied PI3K/AKT pathway [29] stimulates glycolysis by increasing glucose transporter expression and membrane translocation and by phosphorylating the key enzymes of glycolysis [30]. Hypoxia-inducible factor-1a (HIF-1a) increases the glycolytic capacity by promoting the transcription of genes encoding glucose transporters and key enzyme of glycolysis [31]. FLIP_L_ is aberrantly expressed in HCC, and plays an important role in tumor progression [11]. There is evidence showing that FLIP_L_ up-regulates AKT [32] and HIF-1 [19] expression. Considering that AKT/HIF-1 regulate glucose transporter expression, we cannot rule out that FLIP_L_ might regulate glycolysis through the HIF-1 or AKT signaling pathways. Further studies are needed to explore this possibility.

As tumor cells proliferate, they consume large amounts of nutrients, particularly glucose. However, the glucose concentration of cancer tissues is frequently 3-6-fold lower than normal tissue [33]. Therefore, tumor cells must have the capacity to adapt to low levels of nutrients [8]. Aerobic glycolysis is less efficient than oxidative phosphorylation for generating ATP. However, this less efficient ATP generation is beneficial to cell proliferation [34]. Different tumor cells exhibit diverse responses to glucose limitation, and the glucose utilization and mitochondrial function determine the sensitivity to glucose limitation. Increased glucose uptake promotes glycolysis, thereby increasing the capacity to utilize glucose. GLUTs transport glucose into cells depending on the glucose concentration gradient across the cell membrane. In contrast, SGLTs do not rely on the glucose concentration [35]. In prostate cancer, SGLT1 enhanced the survival of tumor cells in low glucose conditions by increasing the intracellular glucose level [36]. We found that SGLT1 also promotes the survival of HCC cells exposed to low glucose levels. Our findings indicate that SGLT1 plays an important role in the adaptation of HCC cells to glucose limitation.

The pathology of HCC is complicated due to the involvement of multiple and distinct signaling pathways in the initiation and development of HCC. This study revealed that FLIP_L_ not only promotes cell survival by modulating cell apoptosis, autophagy, and necrosis [37] but also affects glucose metabolism through the regulation of SGLT1 in HCC. Our findings suggest that FLIP_L_ could be a potential intervention target for HCC therapy in the future.

## Conclusions

In the current study, we provided evidence strongly suggesting that FLIP_L_, an apoptosis suppressor, is positively correlated with SGLT1 expression in HCC. This correlation is associated with FLIP_L_-induced glucose uptake and glucose-dependent aerobic glycolysis. We have further demonstrated that FLIP_L_ plays a significant role in the tolerance ability of HCC to low glucose, during which FLIP_L_ interacts with and protects SGLT1 from proteasomal degradation. Although the contribution of FLIP_L_ to tumor onset and progression is complicated, our findings first identified that FLIP_L_ is involved in HCC energy metabolism and is a promising therapeutic target for future intervention for HCC.

## Additional files

Additional file 1:
**Table S1.** Statistical results for FLIP_L_ expression in the adjacent normal specimens of 79 HCC. **Table S2.** Statistical results of SGLT1 expression in the adjacent normal specimens of 79 HCC. (DOCX 17 kb)
