# Supplementary material for: FLIPL is critical for aerobic glycolysis in hepatocellular carcinoma
Source: J Exp Clin Cancer Res. 2016 May 13;35:79. doi: 10.1186/s13046-016-0358-3 (PMC4865989; doi:10.1186/s13046-016-0358-3)
Supplement: Additional file 1: — Table S1. Statistical results for FLIPL expression in the adjacent normal specimens of 79 HCC. Table S2. Statistical results of SGLT1 expression in the adjacent normal specimens of 79 HCC. (DOCX 17 kb) [file 13046_2016_358_MOESM1_ESM.docx]

**Table S1. Statistical results for FLIP_L_ expression in the adjacent normal specimens of 79 HCC**

| N - +to+++ P-value | | | | |
| --- | --- | --- | --- | --- |
| total | 79 | 60 | 19 |  |
| sex  male  female  Age at diagnosis, years  <60  ≥60 | 66  13  61  18 | 50  10  46  14 | 16  3  15  4 | 1.000^a^  1.000^a^ |

^a^P-value when expression levels were compared using Fisher’ exact test.

**Table S2.** **Statistical results of SGLT1 expression in the adjacent normal specimens of 79 HCC**

| N - +to+++ P-value | | | | |
| --- | --- | --- | --- | --- |
| total | 79 | 69 | 10 |  |
| sex  male  female  Age at diagnosis, years  <60  ≥60 | 66  13  61  18 | 57  12  52  17 | 9  1  9  1 | 1.000^a^  0.440^a^ |

^a^P-value when expression levels were compared using Fisher’ exact test.
